# Accurate 16S Absolute Quantification Sequencing Revealed Vaginal Microecological Composition and Dynamics During Mixed Vaginitis Treatment With Fufang FuRong Effervescent Suppository

**DOI:** 10.3389/fcimb.2022.883798

**Published:** 2022-05-13

**Authors:** Meng Li, Zhen Zeng, Huijun Feng, Yang Cao, Qiongqiong Zhang, Tao Lv, Xingsheng Yang, Dianrong Song, Ping Li, Lina Hu, Shangrong Fan, Ruifang An, Bei Zhang, Lei Zhang, Qinping Liao

**Affiliations:** ^1^ School of Clinical Medicine, Tsinghua University, Beijing, China; ^2^ Department of Obstetrics and Gynecology, Beijing Tsinghua Changgung Hospital, School of Clinical Medicine, Tsinghua University, Beijing, China; ^3^ Department of Obstetrics and Gynecology, Qilu Hospital of Shandong University, Jinan, China; ^4^ Gynecological Department, The First Affiliated Hospital of Tianjin University of Traditional Chinese Medicine, Tianjin, China; ^5^ Department of Obstetrics and Gynecology, The Affiliated Obstetrics and Gynecology Hospital of Nanjing Medical University, Nanjing Maternity and Child Health Care Hospital, Nanjing, China; ^6^ Department of Gynecology, The Second Hospital Affiliated to Chongqing Medical University, Chongqing, China; ^7^ Department of Obstetrics and Gynecology, Peking University Shenzhen Hospital, Shenzhen, China; ^8^ Department of Obstetrics and Gynecology, The First Affiliated Hospital of Xi’an Jiaotong University, Xi’an, China; ^9^ Department of Obstetrics and Gynaecology, Xuzhou Central Hospital, Affiliated Xuzhou Clinical College of Xuzhou Medical University, Xuzhou, China

**Keywords:** accurate 16S absolute quantification sequencing, mixed vaginitis, microbiome, antibiotic, traditional Chinese medicine

## Abstract

**Background:**

The diagnosis and treatment of mixed vaginitis are more complicated than single pathogenic infections, and there may be adverse reactions and several contraindications to conventional antibiotic therapy. Therefore, this study aimed to evaluate the preliminary effects of Fufang Furong Effervescent Suppository for the management of aerobic vaginitis (AV) mixed with bacterial vaginosis (BV) using Accurate 16S absolute quantification sequencing (Accu16S).

**Methods:**

In the present randomized, blind, multi-center clinical trial, women (20 to 55 years) who had received a diagnosis of AV+BV were randomly assigned into clindamycin positive control (n = 41) and Fufang Furong Effervescent Suppository (n = 39) groups. The follow-up occurred in three time periods (V1: -2~0 days; V2: 15-17 days; V3: 40 ± 3 days). At each visit, two vaginal swabs, one for clinical evaluation and one for laboratory examination, were taken from each patient. The Nugent score, Donders’ score, drug-related complications, recurrence rates, and microecological changes of vaginal swabs were assessed in the time three periods.

**Results:**

At baseline, the two groups were similar in frequency of presentation with vaginal burning, odor, abnormal discharge, and itching. No meaningful differences in Nugent and Donders’ scores were detected between the two groups at stage V2 (Nugent: *p* = 0.67; Donders’: *p =* 0.85) and V3 (Nugent: *p* = 0.97; Donders: *p* = 0.55). The Furong group presented fewer complications compared to the Clindamycin group. However, this difference was not statistically significant (*p* = 0.15). Additionally, Accu16S indicated that the total abundance of bacteria in both groups sharply decreased in stage V2, but slightly increased in V3. In stage V3, the absolute abundance of *Lactobacillus* in the Furong group was considerably higher compared to untreated samples (*p* < 0.05). On the other hand, no momentous increase was detected in the Clindamycin group (*p* > 0.05).

**Conclusion:**

Fufang Furong Effervescent Suppository can be as effective as clindamycin cream in the management of AV+BV while may restore the vagina microecosystem better.

## Introduction

Vaginitis is one of the most common causes for women of different ages to visit a health care provider, representing an important concern for public health (Mills, 2017). The most common types of vaginitis affecting women during their reproductive age can be divided based on the pathological agent, including bacterial vaginitis (BV), trichomonal vaginitis (TV), vulvovaginal candidiasis (VVC), and aerobic vaginitis (AV) (Paavonen and Brunham, 2018). Mixed vaginitis refers to the simultaneous presence of at least two vaginal pathogens, both contributing to an abnormal vaginal milieu and the corresponding clinical symptoms and signs (Sobel et al., 2013). Mixed vaginitis is more complicated than single pathogen infections and its prevalence has been poorly documented across the world (Qi et al., 2021). Data from several studies have suggested that BV+TV is the most common mixed vaginitis (37.8%), followed by BV+VVC (14.9%) and BV+VVC+TV (4.1%) (Ozyurt et al., 2001; Rivers et al., 2011). Since the term ‘aerobic vaginitis’ was first introduced by Donders in 2002, increasing studies have shown a high incidence of AV-related mixed vaginitis. Currently, the rate of AV-related mixed infections is the most noteworthy in Chinese women, with 36.9% of AV+BV, 38.1% of AV+VVC, and 25% of AV+trichomoniasis (Fan et al., 2013).

Although the knowledge about mixed vaginitis has increased, its standard treatment has not been standardized yet. Currently, oral metronidazole, vaginal clindamycin cream, or metronidazole gel are the main BV therapies (Donders et al., 2014). The CDC (Centers for Disease Control and Prevention) Sexually Transmitted Infection Treatment Guidelines of 2021 also suggests these treatments (Workowski et al., 2021). For AV, topical vaginal clindamycin or kanamycin or oral other antibacterial drugs that are active against aerobic bacteria such as cefuroxime or moxifloxacin are recommended (Mendling et al., 2016; Wang et al., 2016; Feng et al., 2018). The European (IUSTI/WHO) International Union against sexually transmitted infections (IUSTI) World Health Organisation (WHO) guideline of 2018 also recommends topical clindamycin as the primary treatment for AV (Sherrard et al., 2018). Therefore, clindamycin is also effective in women with mixed AV and BV and was selected as a positive control in the present study.

Women diagnosed with AV+BV would empirically receive antibiotic treatment, which can unbalance the vaginal ecosystem. Additionally, local vaginal burning or irritation and clindamycin resistance (due to the extensive use of antibiotics) have become concerning problems. (Javed et al., 2019). Hence, Traditional Chinese Medicine (TCM) has been considered as an alternative treatment for AV+BV. Clinical research has revealed that the treatment of vaginitis using vaginal lavage with TCM demonstrated satisfactory efficacy (Liu et al., 2014; Li et al., 2016). Treatments for vaginitis based on TCM, such as Fufang Furong Effervescent Suppository, can not only disinfect and relieve itching but also relatively reduce the destruction of the vaginal microbiome. Fufang FuRong Effervescent Suppository (Shaanxi Momentum Qixuehe Pharmaceutical Co., Ltd., China) is comprised of 6 herbs (1000g suppositories): *Sophorae Flavescentis Radix* (320g), *Cnidii Fructus* (230g), *Phellodendri Chinensis Cortex* (110g), *Hibisci Mutabilis Folium* (130g), *Artemisiae Argyi Folium* (120g), *Alumen* (90g).

The advence in high-throughput sequencing has greatly accelerated the study of the vaginal microbiome and gynecological diseases, enabling researchers to map the composition and function of the microbiome in high-resolution and culture-independent models (Almeida and Shao, 2018). However, most of these techniques are relative quantification 16S-seq (RQS) and do not consider absolute bacterial abundances. Inappropriate interpretations based on relative quantifications can mislead some researches (Stämmler et al., 2016).

Thus, accurate absolute quantification of bacterial populations is necessary. Therefore, in the present study, we adopted Accurate 16S absolute quantification sequencing (Accu16S) (Wei et al., 2021) to determine the absolute and relative abundances of the vaginal microbiome and evaluate the preliminary efficacy of Fufang Furong Effervescent Suppository in the treatment of mixed vaginitis.

## Materials and Methods

### Study Design

From August 2019 to June 2021, we conducted a multicenter, randomized, blind, positive parallel controlled trial to assess the efficacy of Fufang Furong Effervescent Suppository for the treatment of AV combined with BV. The trial received ethical approval from the ethics committee of the Beijing Tsinghua Changgung Hospital (19190-0-02). In addition, the protocol was registered in the Chinese Clinical Trial Registry (ChiCTR1900027616). Patients were recruited from gynecological centers of 27 public hospitals in China. In this study, we only analyzed clinical data and vaginal swabs from patients at seven hospitals where specimen preservation conditions were available (distributed in the east, south, west, north and central parts of China), including Peking University Shenzhen Hospital, The First Affiliated Hospital of Xi’an Jiaotong University, Second Affiliated Hospital of Chongqing Medical University, Shandong University Qilu Hospital, Xuzhou Central Hospital, The First Affiliated Hospital of Tianjin University of Traditional Chinese Medicine and Nanjing Maternal and Child Health Care Hospital. The CONSORT Checklist and CONSORT Flow Diagram in supplementary documents. All eligible patients provided written informed consent or legal guardian consent before participating in this study. The first, second, and last authors vouch for the integrity and accuracy of the data and the fidelity of the trial to the protocol.

### Participants

Eligible participants represented women between 20 to 55 years who regularly menstruated and had a sexual history. The inclusion and exclusion criteria are presented in **Table 1**. Currently, the gold standard for BV diagnosis is the Nugent score (Nugent et al., 1991). In this case, a scale of 0-10 is used to assess the disease severity, with 7–10 indicating a positive BV diagnosis. The diagnosis of AV is based on the Donders’ score. The highest AV score is 10, with scores under 3 corresponding to ‘no signs of AV’, 3-4 to ‘light AV’, 5-6 to ‘moderate AV’ and > 6 to ‘severe AV’ (Donders et al., 2017). Only AV patients with light to moderate symptoms were included in this study. Additionally, all participants underwent vaginal microbiota functional tests, such as vaginal pH, H_2_O_2_ concentration, sialidase activity, and leucocyte esterase (LE) activity (Mårdh et al., 2003), as described previously (An et al., 2016). All tests mentioned above were performed three days after menstruation. Furthermore, all qualified patients were also assessed for TCM-related clinical symptoms, including increased vaginal discharge, leucorrhea yellowish or odors. Meanwhile, some basic vital signs and laboratory examinations, such as blood routine, urine routine, liver, and kidney function, were analyzed considering the normal limits.

**Table 1:** Inclusion and exclusion criteria.

Inclusion	Exclusion
Premenopausal women aged 20-55; Sexual history; Conforms to the diagnostic criteria of BV+AV; Donders scores ≥3 and ≤6; Nugent score ≥7; Volunteer to participate and signed an informed consent form;	Combined with other vaginitis such as TV, VVC; Combined with other serious gynecological diseases, such as gynecological malignant tumor and pelvic inflammatory disease; Combined With serious primary diseases of heart, brain, liver, kidney, blood system and endocrine system (ALT, AST≥normal upper limit 2 times;Scr≥normal upper limit); Received any oral or topical medication for the disease within one week; Hypersensitivity to the investigated product or clindamycin; Pregnant and Lactation women or recently planned pregnancy; Combined with neuropsychiatric disorders and suspected or confirmed history of alcohol and drug abuse; Participate in other clinical trials within 1 month;

### Procedures

Patients were randomly assigned into two groups: positive control group (clindamycin phosphate cream - Clindamycin group) and experimental group (Fufang Furong Effervescent Suppository - Furong group). Allocation was based on a random permutation table. The screening period was set as the baseline (-2-0 days - stage V1). Before drug administration, all of the participants were trained to insert vaginal tablets. Then, patients in both groups were put on medication for 12 consecutive days (one tablet once a night), during which they were told to abstain from vaginal intercourse. Next, they were asked to return to the clinic twice, at 3-5 days after the treatment period (15-17 days - stage V2), and 28 ± 3 days after the treatment period (40 ± 3 days - stage V3). At each visit, participants underwent routine laboratory examinations and two vaginal swabs were retrieved, one for assessment of the Nugent and Donders’ scores, and the other was retained at -80°C for further Accu16S. Each participant was assessed for compliance and reported for co-medication and other special circumstances during the period. Moreover, all women were asked to record any side effects of the treatment in a checklist and were asked to bring it in the follow-up visits.

### Accu16STM Assay

Accu16S was performed by Genesky Biotechnologies Inc., Shanghai, 201315 (China). Briefly, total genomic DNA was extracted using TIANamp Bacteria DNA Kit (TIANGEN BIOTECH, Beijing, China) according to the manufacturer’s instructions. The integrity of genomic DNA was detected through agarose gel electrophoresis, and the concentration and purity of genomic DNA were detected through the Nanodrop2000 (Thermo Fisher Scientific, Massachusetts, USA) and Qubit3.0 Spectrophotometer (Thermo Fisher Scientific, Massachusetts, USA). Multiple spike-ins with identical conserved regions to natural 16S rRNA genes and variable regions replaced by random sequence with ~40% GC contents were artificially synthesized. Then, appropriate proportion of spike-ins mixture with known gradient copy numbers were added to the sample DNA. The V3-V4 hypervariable regions of the 16S rRNA gene and spike-ins were amplified with the primers 341F (5^’^-CCTACGGGNGGCWGCAG-3^’^) and 805R (5^’^-GACTACHVGGGTATCTAATCC-3^’^) and then sequenced using Illumina NovaSeq 6000 sequencer (Illumina, California, USA).

### Illumina Read Data Processing and Analysis

The raw read sequences were processed in QIIME2 (Bolyen et al., 2019, 2). The adaptor and primer sequences were trimmed using the cutadapt plugin (Version 0.5.0, Babraham Bioinformatics, UK). DADA2 plugin was used for quality control and to identify amplicon sequence variants (ASVs) (Callahan et al., 2016). Taxonomic assignments of ASV representative sequences were performed at a confidence threshold of 80% by Mothur (Version 1.41.1, https://www.mothur.org/) with the command classify.seqs based on the RDP (Version 11.5) database. Then the spike-in sequences were identified and reads were counted. Standard curve for each sample was generated based the read-counts versus spike-in copy number, and the absolute copy number of each ASV in each sample was calculated by using the read-counts of the corresponding ASV. Since the spike-in sequence is not a component of the sample flora, the spike-in sequence needs to be removed in the subsequent analysis (Jiang et al., 2019).

### Statistical Analyses

Data were gathered at the baseline, 15-17 days, and 40 ± 3 days after treatments. The IBM SPSS 23.0 (IBM Corporation, USA) and R (version 3.5.1) were used for statistical analyses. Descriptive statistics included *N* (%), means, and standard deviations (SD). To compare categorical and quantitative variables between the two groups, χ^2^, and independent *t*-tests were used, respectively. The heatmap, α-diversity, coordinate analysis plots (PCoA), and boxplot figures were constructed using R. A *p* < 0.05 was considered significant.

## Results

### Clinical Data

**Figure 1:**
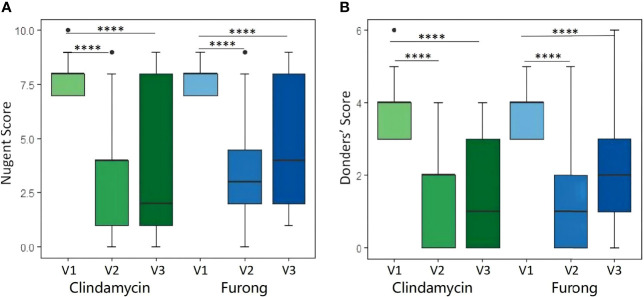
Nugent score **(A)** and Donders score **(B)** before and after Clindamycin and Furong treatments. (****P value < 0.0001).

A total of 80 patients were enrolled from the seven public hospitals mentioned above, and their specific distribution is shown in **Table S1**. No significant differences were detected for the baseline features between the two groups. Moreover, both groups presented a similar rate of vaginal clinical symptoms before therapy (**Table 2**). Clinical efficacy assessment was based on the Nugent and Donders’ scores during follow-up. The prognosis of AV and BV in both groups was notably improved (**Figure 1**). The comparable and sharp decline of the Nugent score is shown in **Figure 1A**. Besides, most patients presented an abrupt decline in their Donders’ scores (**Figure 1B**). No significant differences were detected for the Nugent and Donders’ scores between the two groups at each follow-up period (**Tables 3**, **4**). In stage V2, 20 patients (51.3%) in the Clindamycin group were defined as BV-negative (Nugent 0-3) and 12 (30.8%) as BV intermediate (Nugent 4-6). In the Fufang Furong Effervescent Suppository group, 19 (48.7%) patients were BV-negative, and 13 (33.4%) were BV intermediate (*p* = 0.67). In stage V3, 16 (48.5%) patients in the Clindamycin group were BV-negative and 4 (12.1%) were BV intermediate. In the Furong group, 19 (54.3%) patients were BV-negative and 3 (8.6%) were BV intermediate (*p* = 0.97) (**Table 3**). Only 9 patients in both groups were diagnosed with mild to moderate AV at stage V2 (*p* = 0.85; **Table 4**). At stage V3, 9 mild and 2 moderate AV patients were identified in the Furong group. Meanwhile, only 10 patients in the Clindamycin group had mild AV. However, this difference between the treatment groups was not statistically significant.

**Table 2:** Sample characteristics and vaginal clinical symptoms at the baseline.

Variable	Clindamycin Group (n=41)	FuRong Group (n=39)	P value
**Characters (mean ± SD)**			
Age (years)	37.07 ± 8.74	35.67 ± 8.36	0.46
Body mass index (kg/m^2^)	21.05 ± 2.59	21.77 ± 3.46	0.30
Pregnancy	2.17 ± 1.52	2.38 ± 1.53	0.53
Parity	1.15 ± 0.85	1.21 ± 0.73	0.74
**Clinical symptoms n, (%)**			0.65
Itching	11 (26.8)	13 (33.3)	
Bad odor	20 (51.3)	24 (61.5)	
Burning	2 (5.13)	3 (7.6)	
Abnormal vaginal discharge	30 (73.2)	30 (76.9)	
**Diagnosed First Time n, (%)**			0.33
Yes	38 (92.7)	38 (97.4)	
No	3 (7.3)	1 (2.6)	

**Table 3:** Nugent score during the three follow-up periods.

Nugent Score	Clindamycin Group (n=41)	FuRong Group (n=39)	P value
**V1 n, (%)**			0.19
7	15 (36.6)	15 (38.5)	
8	20 (48.8)	23 (59.0)	
9	3 (7.3)	1 (2.6)	
10	3 (7.3)	0	
**V2 n, (%)**			0.67
0-3	19 (48.7)	20 (51.3)	
4-6	13 (33.4)	12 (30.8)	
7	1 (2.6)	4 (10.3)	
8	5 (12.8)	1 (2.6)	
9	1 (2.6)	2 (5.1)	
**V3 n, (%)**			0.97
0-3	19 (54.3)	16 (48.5)	
4-6	3 (8.6)	4 (12.1)	
7	3 (8.6)	1 (3.0)	
8	9 (25.7)	10 (30.3)	
9	1 (2.9)	2 (6.1)	

**Table 4:** Donders’ score during the three follow-up periods.

Donders’ Score	Clindamycin Group (n=41)	FuRong Group (n=39)	P value
**V1 n, (%)**			0.63
3-4	33 (80.5)	33 (84.6)	
5-6	8 (19.5)	6 (15.4)	
**V2 n, (%)**			0.85
<3	30 (76.9)	30 (76.9)	
3-4	9 (23.1)	8 (20.5)	
5-6	0	1 (2.6)	
**V3 n, (%)**			0.55
<3	25 (71.4)	22 (66.7)	
3-4	10 (28.6)	9 (27.3)	
5-6	0	2 (6.0)	

The relapse rate was also diagnosed based on the Nugent and Donders’ scores (**Table 5**). The Clindamycin group had fewer relapse patients than the Furong group, but it was not significant (AV: *p* = 0.22; BV: *p* = 0.20). On the other hand, more patients in the Clindamycin group did not respond to the treatment compared to the Furong group but without any substantial difference (AV: *p* = 0.18; BV: *p* = 0.26). Then, we analyzed treatment side effects in these two groups. The side effects reported mainly involved VVC, urticaria, vaginal burning, and liver function damage. In the Clindamycin group, one patient developed severe urticaria 1 h after medication, which was later improved by antiallergic therapy. Adverse reactions occurred in 9 (22.0%) patients in the Clindamycin group and 7 (17.9%) in the Furong group (*p* = 0.65). Among them, 6 (14.6%) cases in the Clindamycin group and 2 (5.1%) in the Furong group had drug-related complications, but this difference was not significant (*p* = 0.15). Two patients who developed severe VVC after clindamycin treatment were excluded from the follow-up due to antifungal therapy.

**Table 5:** Recurrence and persistence 28 ± 3 days after medications.

	Clindamycin Group (n=41)	FuRong Group (n=39)	P value
**Relapse**			
AV	6	10	0.22
BV	5	9	0.20
**Persist**			
AV	4	1	0.18
BV	5	2	0.26

### Absolute Quantification of Vaginal Swabs

**Figure 2:**
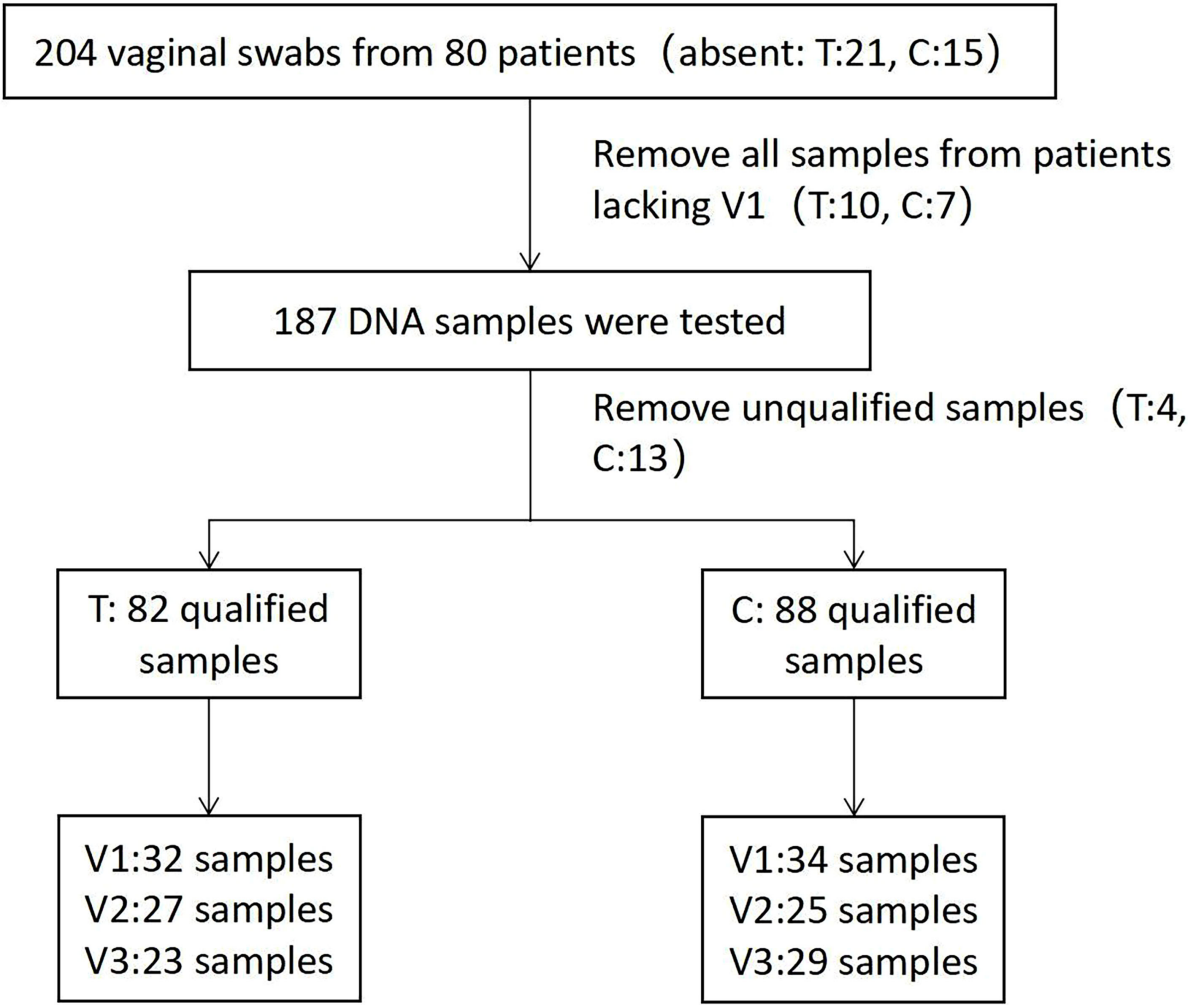
Flowchart of Accu16S qualified sample screening.

**Figure 3:**
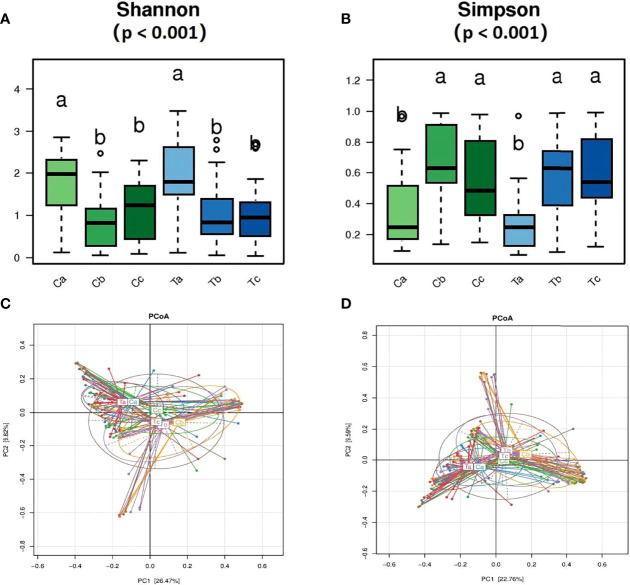
Comparisons of the vagina microbiota α diversity **(A, B)** and relative **(C)** and absolute **(D)** abundances in the principal coordinate analysis (PCoA) of bacterial β diversity. **(A, B)** rank all the mean values from highest to lowest using significant difference letter notation. The largest mean is marked with the letter a and compared with other means, where the difference is not significant, the letter a is marked until an average with a significant difference is marked with the letter b.

A total of 170 qualified samples were used for Accu16S (**Figure 2**). After the removal of ambiguous, short, low-quality reads and singleton OTUs, a total of 34108020 reads remained for community analysis of 170 samples. Spike-in reads accounted for 27.67 ± 9.0% (18.67-36.67%) of total reads in a given library, similar to the expected proportion of 30%. The α-diversity indicators Shannon and Simpson (Both of them are commonly used to reflect α-diversity. The larger the Shannon value, the higher the community diversity. The greater the Simpson index value, the lower the community diversity.) for the vagina microbiota in both groups are shown in **Figures 3A, B**. The diversity of bacteria in these samples abruptly decreased compared to initial untreated samples (*p* = 0.00). The PCoA based on weighted Unifrac matrix clustering data of microbial β-diversity among the 170 samples is shown in **Figure 3C** (relative abundance) and 3D (absolute abundance). Vaginal samples from stage V1 in both groups were gathered and separated from those in stages V2 and V3. Moreover, the vaginal microbiota composition was similar in stages V2 and V3, similar to the clinical data. The absolute and relative quantification did not influence the PCoA.

**Figure 4:**
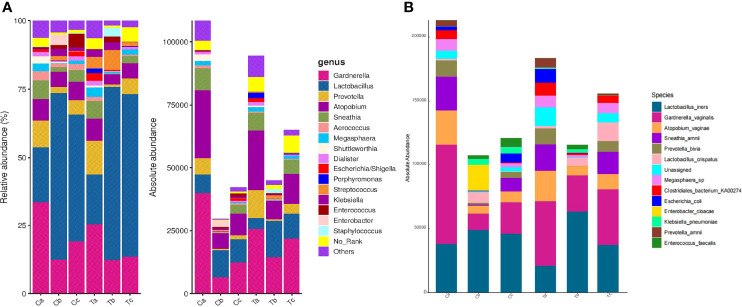
Relative and absolute abundances of the major taxa at the genus **(A)** and the species **(B)** levels in Clindamycin and Furong groups.(Ta, Tb, and Tc represent stages V1, V2, and V3 of the Furong group, respectively; Ca, Cb, and Cc represent stages V1, V2, and V3 of the Clindamycin group, respectively).

**Figure 5:**
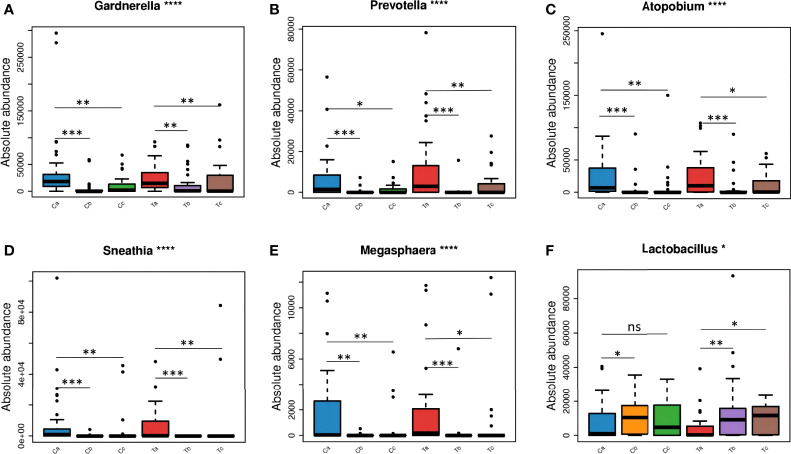
The changes of dominant microorganisms between the two groups at each visit: *Gardnerella* **(A)**, *Prevotella* **(B)**, *Atopobium* **(C)**, *Sneathia* **(D)**, *Megasphaera* **(E)**, and *Lactobacillus* **(F)**. (Ta, Tb, and Tc represent stages V1, V2, and V3 of the Furong group, respectively; Ca, Cb, and Cc represent stages V1, V2, and V3 of the Clindamycin group, respectively; ns, nonsense; *0.01≤P value < 0.05; **0.001≤P value < 0.01; *** 0.0001≤P value < 0.001; ****P value < 0.0001).

After treatment, the total abundance of bacteria in both groups sharply decreased in stage V2, but slightly increased in V3, especially in the Furong group (**Figure 4A**). The maximal loss of the total bacteria amount was detected in stage V2 of clindamycin treatment. *Gardnerella*, *Prevotella*, *Atopobium, Sneathia*, and *Megasphaera* were the dominant bacteria before the treatment (**Figure 4A**). The absolute quantification of these five bacteria without treatment did not differ between the two groups and harshly decreased after treatment (**Figure 5**). Despite the strong sterilization promoted by clindamycin, the absolute abundance of *Lactobacillus* in the vagina was still slightly increased. Interestingly, in the Furong group, the absolute abundance of *Lactobacillus* also notably increased while pathogenic bacteria diminished. The relative abundance of dominant bacteria in stage V1 was similar to their absolute abundance in both groups (**Figure 4A**). However, RQS considerably amplified the quantity of *Lactobacillus* in stages V2 and V3, which might mislead the judgment. The comparison between relative and absolute quantifications of several important bacterial communities during the entire course of treatment is presented in **Figure S1**. Overall, the relative quantification can reflect the changing trend of bacteria to a certain extent, but it can distort the therapeutic effects due to inaccurate quantification.

**Figure 6:**
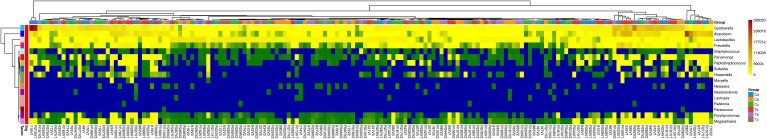
Heatmap of the distributions of the 17 most abundant bacterial genera present in the vaginal swab samples. (The abscissa represents the sample, the ordinate represents different species at the genus level. The absolute abundances of bacterial genera are indicated by color intensity. Ta, Tb, and Tc represent stages V1, V2, and V3 of the Furong group, respectively; Ca, Cb, and Cc represent stages V1, V2, and V3 of the Clindamycin group, respectively).

Three types of bacteria (*Gardnerella*, *Prevotella*, and *Atopobium*) had a faint increment during stage V3 in both groups (**Figure 4A**). Therefore, we conducted a Wilcoxon rank-sum test for these bacteria in stages V1 and V3 for each group. The absolute abundance of pathogenic bacteria in stage V3 was greatly lower compared to before either Clindamycin or Furong treatments (**Figure S2**). Meanwhile, the absolute abundance of *Lactobacillus* in the Furong group was considerably higher compared to untreated samples (*p* < 0.05) but did not differ from the Clindamycin group (*p* > 0.05). Since 16S analyses can not efficiently classify the annotation results at the species level, we manually compared the sequences in NCBI (National Center for Biotechnology Information to obtain the results in **Figure 4B**. In both groups, *Lactobacillus iners* was dominant in *Lactobacillus* spp., before or after treatments. Its changes were similar to the overall trend of *Lactobacillus*, of which both showed an increase in the total amount after the treatment, especially in the Furong group. Another important increase in *Lactobacillus* species was represented by *Lactobacillus crispatus.* Interestingly, *L. crispatus* only had a transient increase in stage V2 in the Clindamycin group and did not differ 28 ± 3 days after management. Meanwhile, *L. crispatus* continued to increase after Furong therapy with a measurable centrality (*p* < 0.05). Furthermore. the species level analyses (**Figure 4B**) confirmed *Gardnerella vaginalis, Atopobium vaginae, Sneathia amnii*, and *Prevotella bivia* as the dominant pathogenic bacteria. At the genus level (heatmap - **Figure 6**), a cluster pattern of the bacterial community composition was identified, similar to the grouping pattern observed using PCoA. First, *Lactobacillus* became the dominant bacteria in vaginal microbiota at stages V2 and V3 in both groups. Second, a preponderance of *Gardnerella, Prevotella*, and *Atopobium* was detected among pre-treatment patients, consistent with our previous findings.

## Discussion

Vaginal infections, such as BV, TV, VVC, and AV, affect the health and quality of life of women and can lead to adverse gynecological complications and reproductive outcomes (Marnach et al., 2022). Several reports have shown that incident BV is associated with an initial decrease in the number of healthy-associated *Lactobacillus* species *(L.crispatus, L.iners, L.jensenii*, and *L.gasseri)* and a subsequent increase in the abundance of *Gardnerella*, *Prevotella*, *Atopobium*, and other anaerobic bacteria (Muzny et al., 2018), consistent with our current results. At the species level, *L.iners* was the dominant *Lactobacillus* in AV+BV patients, and the main pathogenic bacteria were *Gardnerella vaginalis, Atopobium vaginae, Sneathia amnii*, and *Prevotella bivia* (**Figure 4B**), similar to previous studies (Muzny et al., 2018). BV is characterized by dysbiosis of the vaginal microbiota and often occurs with another vaginitis, especially TV and AV. In China, due to the high incidence of combined AV and BV(accounted for 65.35% in mixed vaginitis), more attention should be directed to these infections (Wang et al., 2022). Although AV and BV share some similarities, such as a decrease or absence of *Lactobacillus*, an increase in vaginal secretions, odor, and pH increment (usually more pronounced in AV), there are meaningful differences between them. Different from BV patients, AV patients have increased aerobic bacteria, mostly *Escherichia coli*, *group B streptococci*, and *Staphylococcus aureus* (Donders et al., 2017; Wang et al., 2020). In our current study, no notably changes related to these aerobic bacteria were observed, which might be related to the fact that most patients recruited had light, or mild to moderate AV (**Table 4**). Clindamycin is active against staphylococci and streptococci as well as anaerobes. Antibiotics are a vital resource for treating genital infections, but the rise of antibiotic-resistant bacteria has become a public health problem. Due to antibiotic resistance, 30% recurrence rate for BV occurs within the first month, 59% within 6 months, and 69% within 12 months of treatment (Bradshaw et al., 2006). Moreover, many AV-related bacteria have developed clear clindamycin resistance (Asghar et al., 2020; Genovese et al., 2020). The reduced susceptibility to antibiotics in recent years raised concerns about their use as alternatives. Traditional Chinese Medicine is an important part of Complementary and Alternative Medicine. TCM has been widely used to prevent and treat diseases in China and other parts of East Asia for thousands of years (Zhang et al., 2019). For the past few years, there have been increasing studies regarding the application of TCM in the treatment of genital infections (Zhao et al., 2021). In the TCM theory, vaginitis is usually classified as the area of “leukorrheal diseases,” and its causative pathogens can be classified as heat, damp, cold, and toxin (Lin et al., 2019). Generally, TCM treatment of gynecological infections can be used in two ways: oral and external use, which can be applied independently or in combination. The Fufang Furong Effervescent Suppository is a vaginal topical medication, which encompasses a great therapeutic effect on vaginitis. Its main component, *Sophora flavescens*, has many effects, including antibacterial and anti-inflammatory, insecticidal and anti-pruritic, anti-viral, and analgesic (He et al., 2015). Many studies have confirmed that the pharmacological mechanism of *sophora flavescens* may be *via* the activation or suppression of key molecules in certain cellular signaling pathways, such as nuclear factor kappa B (NF-κB), phosphatidylinositol 3-kinase/protein kinase B/mammalian target of rapamycin (PI3K/AKT/mTOR) and transforming growth factor-β/mothers against decapentaplegic homolog (TGF-β/Smad) (Lin et al., 2022; Tang et al., 2022). Furthermore, other herbs in the formula also have certain bactericidal and anti-inflammatory effects, which complement each other. In the present study, we demonstrated the effect of Fufang Furong Effervescent Suppository in the treatment of AV+BV and that it did not differ from clindamycin. No significant differences in the cure rates were detected between the two groups at 3-5 and 28 ± 3 days after BV treatment (**Table S2**). For AV, there was also no significant difference in scores between the two groups at stages V2 and V3. Overall, no discrepancy was observed in the treatment effect of AV+BV between the two groups.

Drug-related complications occurred in six patients in the Clindamycin group (VVC occurred in three cases, liver damage in one case, severe allergic reaction in one case, and vulvar irritation in one case) and two patients in the Furong group (one case of vaginal burning and one of contact bleeding). Probably due to the small sample size, no remarkable difference in adverse drug reactions was observed between these two groups. Although the number and extent of complications caused by antibiotics were much more severe than those caused by the Furong treatment, we need a larger volume of data to verify these hypotheses. The most interesting aspect of this study was that 28 ± 3 days after the end of treatment, the recovery of the vaginal microbiome in the Furong group was better than that in the Clindamycin group (**Figures S2D–H**). This corresponded to the minimum amount of absolute quantitative bacteria in stage V2 in the Clindamycin group (**Figure 4A**), indicating that clindamycin has a strong bactericidal ability and a big destructive force to the vaginal microbiome, finally requiring more time for vaginal microbiome recovery. This was better verified at the species level: *L. crispatus* is widely accepted as a mainstay in maintaining vaginal microecological balance in healthy women and we found that it significantly increased at stage V3 in the Furong group (**Figure 4B**). Recently, the studies on the faster recovery of vaginal flora have mainly focused on the addition of probiotics during or after treatment (Ho et al., 2016; Superti and De Seta, 2020). However, the effectiveness of adding probiotics remains controversial. A randomized controlled trail showed that oral probiotic *Lacticaseibacillus rhamnosus GR-1* and *Limosilactobacillus reuteri RC-14* adjunctive treatment did not increase the cure rate of BV patients compared to metronidazole alone (Zhang et al., 2021). Another meta-analysis indicated that probiotics used after antibiotic treatment were only effective in the short term (Wang et al., 2019). In the best scenario, TCM replacement therapy can restore the vaginal flora quickly while the therapeutic effect is not inferior to antibiotics.

Moreover, RQS can reflect the proportion and trends of microorganisms to some extent, but it might generate spurious results and can not reveal the variation in absolute abundances of individual microrganisms (Props et al., 2017; Guo et al., 2020). Comparing microbial abundances between different samples in various temporal dimensions is important in the prognosis and clinical medication guidance. The RQS also provides a deep insight into the composition of bacterial communities in vaginal swabs. However, it can not provide dynamic information on the amount of bacterial DNA before and after treatments with different medications (**Figure 4**). More recently, Tkacz et al. (2018) developed absolute quantitation of amplicon families using synthetic chimeric DNA spikes, which has been shown to uncover the comprehensive dynamics of bacterial communities (Jiang et al., 2019). Here, we aimed to use absolute quantification to identify the therapeutic effect and compare it with relative quantification. Both quantitative methods showed that the three pathogenic bacteria closely related to BV were slightly elevated in stage V3 (**Figure 4**), consistent with the fact that BV is prone to relapse in clinical practice (Bostwick et al., 2016). Nevertheless, absolute quantification more accurately displayed the trend of pathogenic bacteria and could better predict recurrence. In the present study, the proportion of *Lactobacillus* was considerably increased in both groups after treatment by relative qualification, which was inconsistent with the absolute data. This apparent relative increase in the proportion of *Lactobacillus* was caused by a decrease in pathogenic bacteria such as *Gardnerella*, *Prevotella*, *Atopobium*. Similarly, relative quantification also overstates the proportion of pathogenic bacteria compared to absolute quantification in stages V2 and V3, which might affect the judgment regarding medication efficacy (**Figure S1**). Additionally, both absolute and relative quantifications showed an outstanding increase of *Lactobacillus* in the vagina after 28 ± 3 days after Furong treatment (**Figures S3A, B**). However, in the Clindamycin group, the absolute quantification indicated that the total amount of *Lactobacillus* in stage V3 did not significantly change (*p* > 0.05), while the overall sum of *Lactobacillus* under relative quantification was notably higher compared to untreated samples (*p* < 0.05) (**Figures S3C, D**). From the absolute quantification, we can conclude that Fufang Furong Effervescent Suppository seems to be better restore the vaginal microbiome than clindamycin, but the relative quantification covered this result. Altogether, these results indicated that relative quantification can sometimes obscure important results and lead to improper conclusions.

Our study also has some limitations. First, the integrity of the experiment was compromised by the loss of some vaginal swabs and unqualified samples during follow-up. Second, Accu16S does not have sufficient resolution to identify microbiomes at the species or strain level, which might result in the omission of some microbial taxa. In the future, quantitative metagenomic sequencing might be a good alternative to better understand the dynamics of the microbiome during disease treatment.

## Conclusion

Overall, the Nugent and Donders’ scores improved in Furong and Clindamycin groups after treatment, and no significant difference was detected between the two groups regarding the theraputic efficiency. Moreover, Accu16S was superior to RQS to accurately assess the dynamics of the vaginal microbiome and evaluate drug effects during treatment. Finally, we demonstrated that Fufang Furong Effervescent Suppository has the same therapeutic effect as clindamycin in the treatment of AV+BV, and may restore the vaginal microecology better.

## Data Availability Statement

The datasets presented in this study can be found in online repositories. The names of the repository/repositories and accession number(s) can be found below: https://www.ncbi.nlm.nih.gov/, PRJNA809916.

## Ethics Statement

The studies involving human participants were reviewed and approved by the ethics committee of Beijing Tsinghua Changgung Hospital (19190-0-02). The patients/participants provided their written informed consent to participate in this study.

## Author Contributions

QL and LZ designed and conducted the research. QZ, TL, XY, DS, PL, LH, SF, RA and BZ carried out the clinical trail. ML, HF, and YC analyzed the data and wrote the paper. ZZ provided critical revisions of the article for intellectual content. All authors contributed to the article and approved the submitted version.

## Funding

This trial was funded by National Key Research and Development Program (2018YFC1707404), Beijing Natural Science Foundation (7202239), Beijing Traditional Chinese Medicine Science and Technology Development Fund Project (JJ-2020-09), Ministry of Science and Technology of China Biotechnology Development Center, National Key Research and Development Program of TCM Modernization Research key project (2018YFC1707410-04).

## Conflict of Interest

The authors declare that the research was conducted in the absence of any commercial or financial relationships that could be construed as a potential conflict of interest.

## Publisher’s Note

All claims expressed in this article are solely those of the authors and do not necessarily represent those of their affiliated organizations, or those of the publisher, the editors and the reviewers. Any product that may be evaluated in this article, or claim that may be made by its manufacturer, is not guaranteed or endorsed by the publisher.
