# Supplementary material for: Accurate 16S Absolute Quantification Sequencing Revealed Vaginal Microecological Composition and Dynamics During Mixed Vaginitis Treatment With Fufang FuRong Effervescent Suppository
Source: Front Cell Infect Microbiol. 2022 May 13;12:883798. doi: 10.3389/fcimb.2022.883798 (PMC9136393; doi:10.3389/fcimb.2022.883798)
Supplement: Supplementary file 1 [file DataSheet_1.pdf]

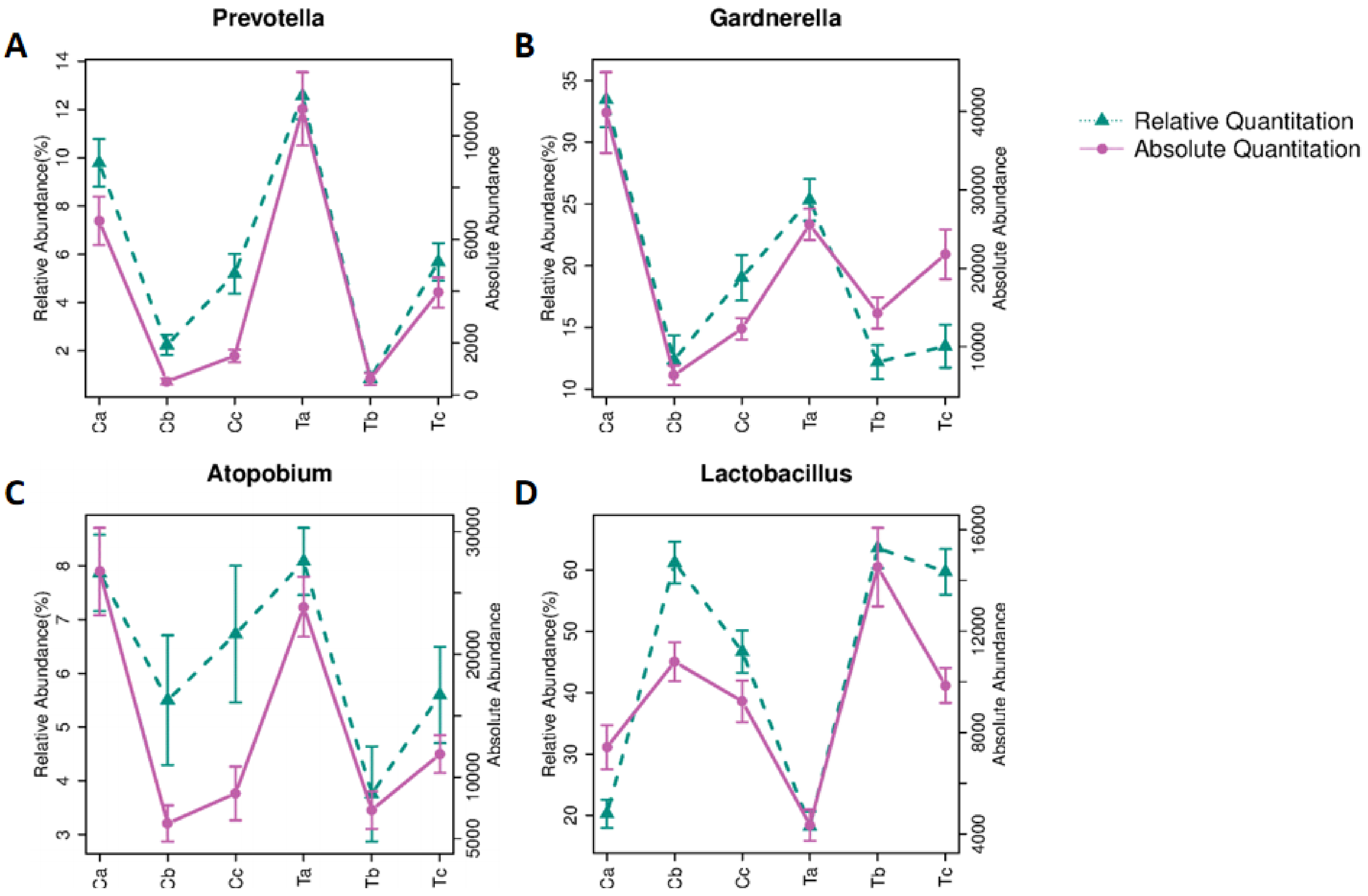

Figure S1. Comparison of relative and absolute quantification in Prevotella (A), Gardnerella (B), Atopobium (C), and Lactobacillus (E). (Ta, Tb, and Tc represent stages V1, V2, and V3 of the Furong group, respectively; Ca, Cb, and Cc represent stages V1, V2, and V3 of the Clindamycin group, respectively)

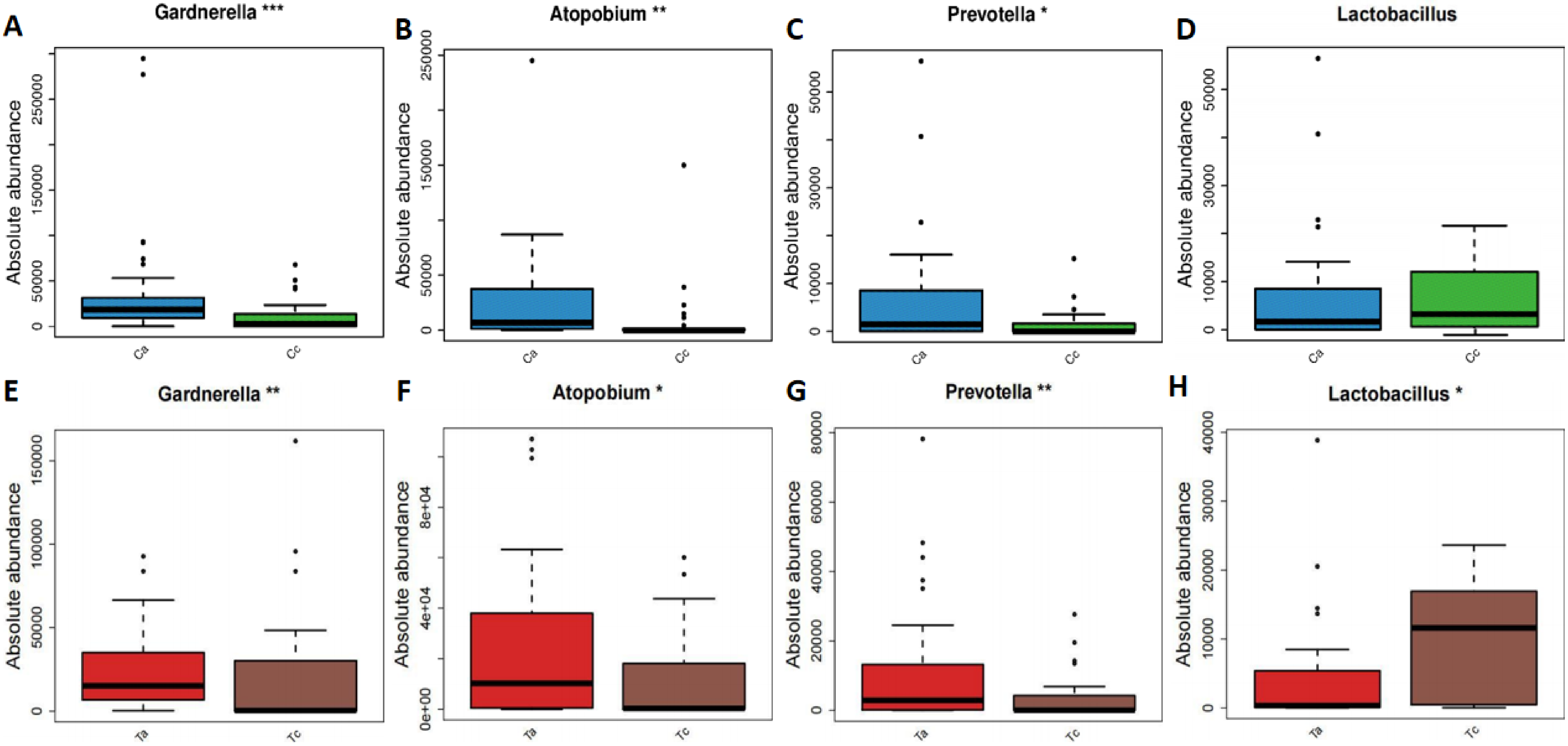

Figure S2. Absolute quantification of several vital bacteria at stage V1 and V3 in Clindamycin group and Furong group. (*0.01≤P value < 0.05; **0.001≤P value < 0.01; *** 0.0001≤P value < 0.001; ****P value < 0.0001;Ta and Tc represent stages V1and V3 of the Furong group, respectively; Ca Cc represent stages V1 and V3 of the Clindamycin group, respectively).

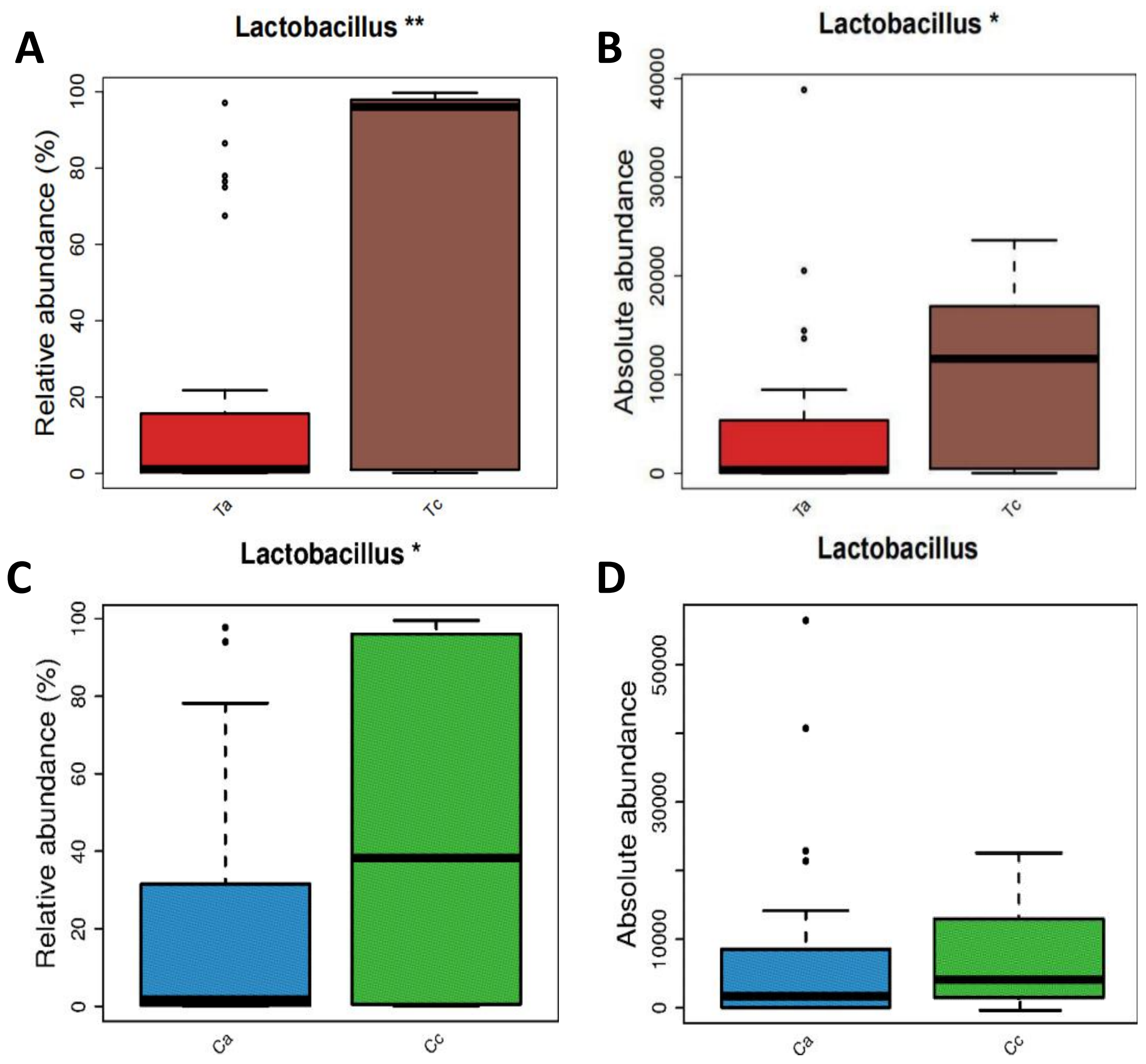

Figure S3. Comparison of relative and absolute quantification of Lactobacillus in Furong Group (A, B) and Clindamycin Group (C, D). (Ta and Tc represent stages V1 and V3 of the Furong group, respectively; Ca and Cc represent stages V1 and V3 of the Clindamycin group, respectively ; **$0.001 \leq P$ value < 0.01; *$0.01 \leq P$ value < 0.05)
