# Supplementary material for: Accurate 16S Absolute Quantification Sequencing Revealed Vaginal Microecological Composition and Dynamics During Mixed Vaginitis Treatment With Fufang FuRong Effervescent Suppository
Source: Front Cell Infect Microbiol. 2022 May 13;12:883798. doi: 10.3389/fcimb.2022.883798 (PMC9136393; doi:10.3389/fcimb.2022.883798)
Supplement: Supplementary file 2 [file DataSheet_2.pdf]

Table S1 Distribution of patients from 7 hospitals

Hospital	Clindamycin Group	FuRong Group	Total
Peking University Shenzhen Hospital	3(7.3)	4(10.3)	7(8.8)
The First Affiliated Hospital of Xi 'an Jiaotong University	3(7.3)	2(5.1)	5(1.3)
Second Affiliated Hospital of Chongqing Medical University	6(14.6)	6(15.4)	12(15.0)
Shandong University Qilu Hospital	10(24.4)	11(28.2)	21(26.3)
Xuzhou Central Hospital	3(7.3)	1(2.5)	4(5.0)
The First Affiliated Hospital of Tianjin University of Traditional Chinese Medicine	8(19.5)	9(23.1)	17(21.3)
Nanjing Maternal and Child Health Care Hospital	9(22.0)	6(15.4)	14(17.5)
Total	41(100)	39(100)	80(100)

Data presented as n (%)

Table S2 Diagnosis during the follow-up period

Diagnosis	Clindamycin Group (n=41)	FuRong Group (n=39)	P value
**V2**			0.67
Single AV	6	5	
Single BV	4	3	
AV+BV	3	4	
**V3**			
Single AV	1	3	0.34
Single BV	4	5	
AV+BV	9	8	
