# Supplementary figures and images for: Accurate 16S Absolute Quantification Sequencing Revealed Vaginal Microecological Composition and Dynamics During Mixed Vaginitis Treatment With Fufang FuRong Effervescent Suppository
Source: Front Cell Infect Microbiol. 2022 May 13;12:883798. doi: 10.3389/fcimb.2022.883798 (PMC9136393; doi:10.3389/fcimb.2022.883798)

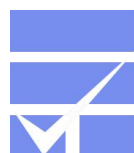

## CONSORT 2010 Flow Diagram

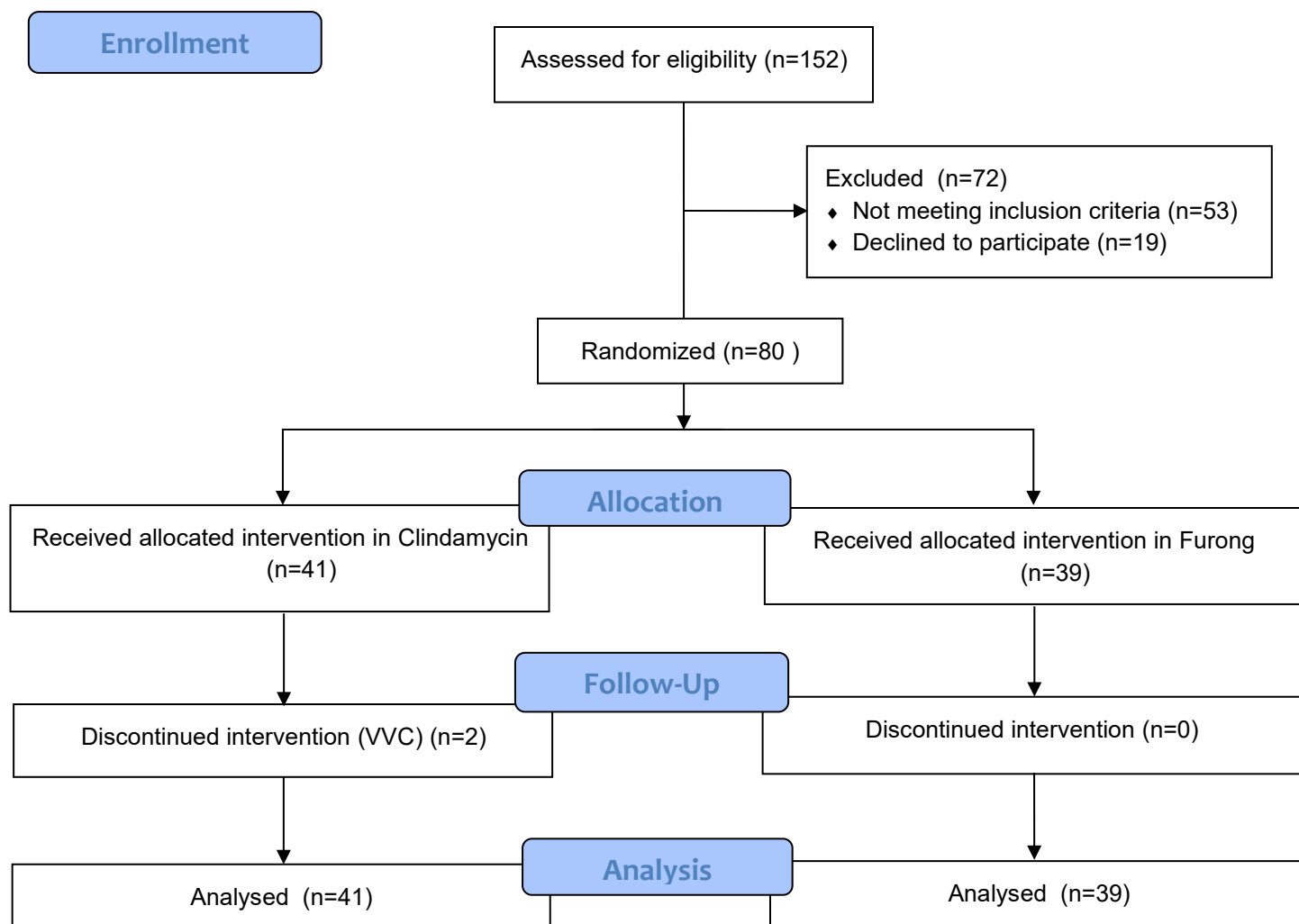

Supplement: Supplementary file 4 [file DataSheet_4.pdf]
